# Single-site treatment of cystic intestinal duplication malformations via the umbilicus

**DOI:** 10.3389/fsurg.2025.1629215

**Published:** 2025-08-18

**Authors:** Tian Xia, Hong Qin, BingQiang Tang

**Affiliations:** Department of General Surgery, Children's Hospital Affiliated to Shandong University, Jinan, Shandong, China

**Keywords:** intestinal duplication deformity, children, intestinal duplication deformity excision, bowel resection and anastomosis, diagnosis, treatment

## Abstract

**Objective:**

To summarize the diagnosis and treatment experience of small intestinal duplication malformations in our hospital.

**Methods:**

We retrospectively analyzed data from 90 children undergoing surgery for intestinal duplication malformations at our hospital from October 2019 to October 2024. All patients underwent transumbilical single-site laparoscopic-assisted resection. A 1.5 cm longitudinal umbilical incision was made, followed by layered dissection of the skin and subcutaneous tissue. Two 5 mm trocars were placed at the incision edges to establish CO_2_ pneumoperitoneum. Bowel graspers were inserted to locate lesions under direct vision. First, the abdominal cavity was examined. The intestinal tube was initially checked for any mass, adhesion, or obvious congestion and edema. This is mostly where the lesion is located. If it is not found, retrograde exploration of the small intestine begins from the ileocecal area. After identifying the mass, it was fixed with absorbable sutures. The trocars and laparoscope were removed, the fascia and peritoneum were incised, and the mass with attached bowel was exteriorized through the umbilical incision. Based on lesion characteristics, enucleation of the duplication cyst or bowel resection with anastomosis was performed. Specimens were sent for pathology.

**Results:**

All surgeries succeeded (duration: 45–95 min). Oral intake resumed within 1–4 days, and discharge occurred at 5–14 days postoperatively. Enucleation was performed in 74 cases, while bowel resection with anastomosis was required in 16 cases (including 5 terminal ileal resections with anastomosis 1.5–2 cm from the ileocecal valve). No complications (incisional infection, anastomotic leakage, stenosis, adhesive bowel obstruction, or incisional hernia) occurred. Pathology confirmed intestinal duplication malformations.

**Conclusion:**

Intestinal duplication malformations predominantly affect the terminal ileum. Enucleation is optimal, but resection-anastomosis is safe when enucleation is difficult, avoiding enterostomy and reducing patient discomfort. Single-site laparoscopy offers minimal invasiveness and excellent cosmetic outcomes.

## 1 Introduction

Intestinal duplication is a rare congenital digestive tract anomaly, predominantly occurring in the small intestine, especially the ileum (1, 2). Pathologically, it is classified into cystic (≈80%) and tubular types. Cystic subtypes include extraluminal and intraluminal variants; the latter may cause early obstruction. Tubular-type duplications are located at the mesenteric attachment border and course parallel to the normal intestine, forming a double-barreled lumen. Intestinal duplication malformations may be lined with gastric mucosa or ectopic pancreatic tissue. The clinical symptoms differ based on the type and location of the lesion as well as the child's age. Common presentations include intestinal obstruction, gastrointestinal bleeding, intestinal necrosis, and peritonitis. Diagnosis is typically achieved using ultrasound, CT, radionuclide scan (ECT), and other modalities. The common differential diagnoses are mesenteric cysts, omental cysts, ovarian cysts, Meckel's diverticulum, etc. Surgical intervention is indicated upon diagnosis of intestinal duplication malformations. Currently, driven by advancements in medical technology, laparoscopic minimally invasive surgery is routinely employed, with transumbilical single-site laparoscopy emerging as the predominant surgical approach. This study aims to synthesize treatment experiences by conducting a retrospective analysis of clinical data from children with intestinal duplications treated at our institution.

## 2 Data and methods

### 2.1 General methods

**Figure 1:**
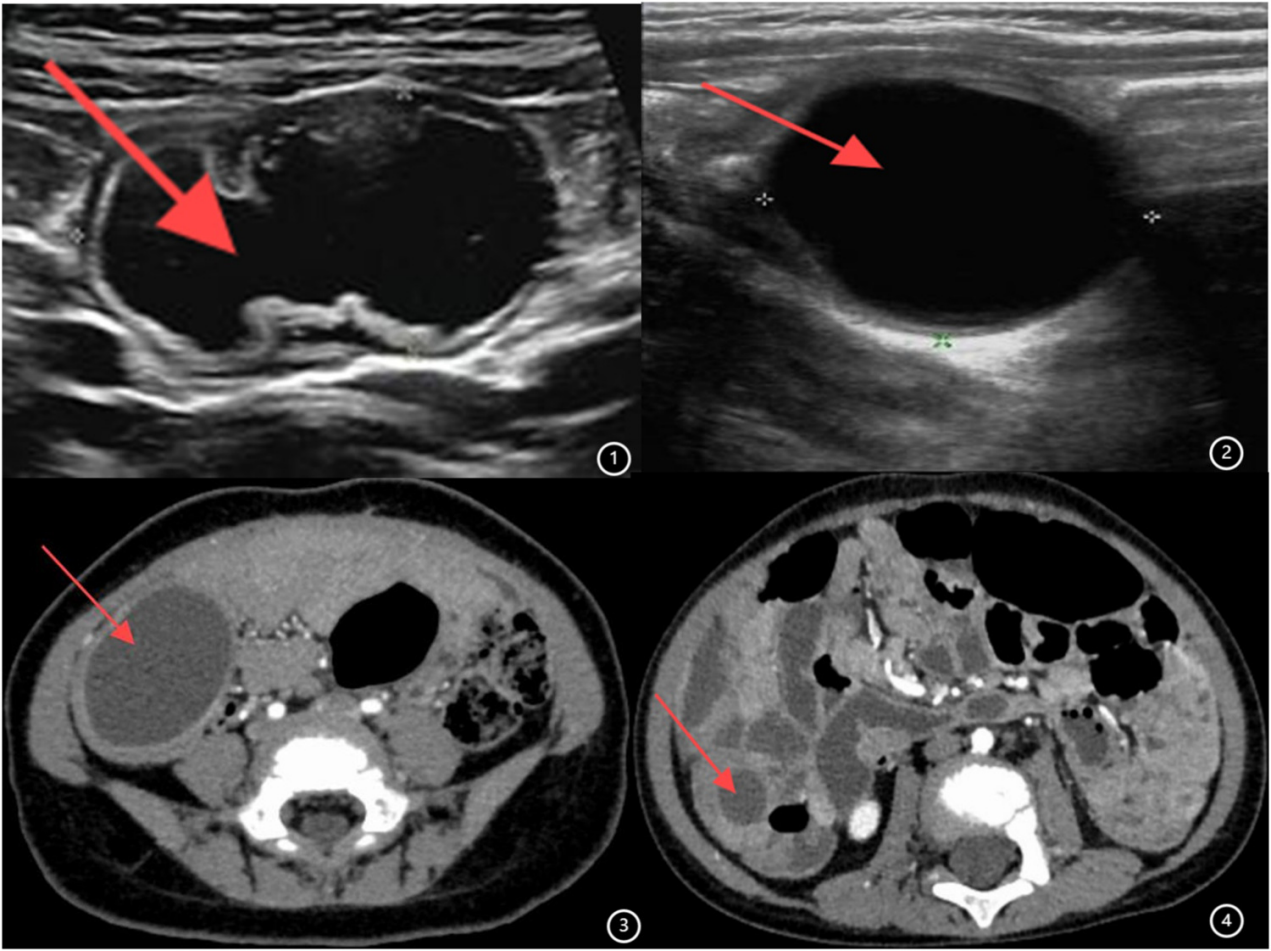
**(1,2)** Ultrasound image of intestinal duplication malformation. **(3,4)** Enhanced CT image of intestinal duplication malformation.

**Figure 2:**
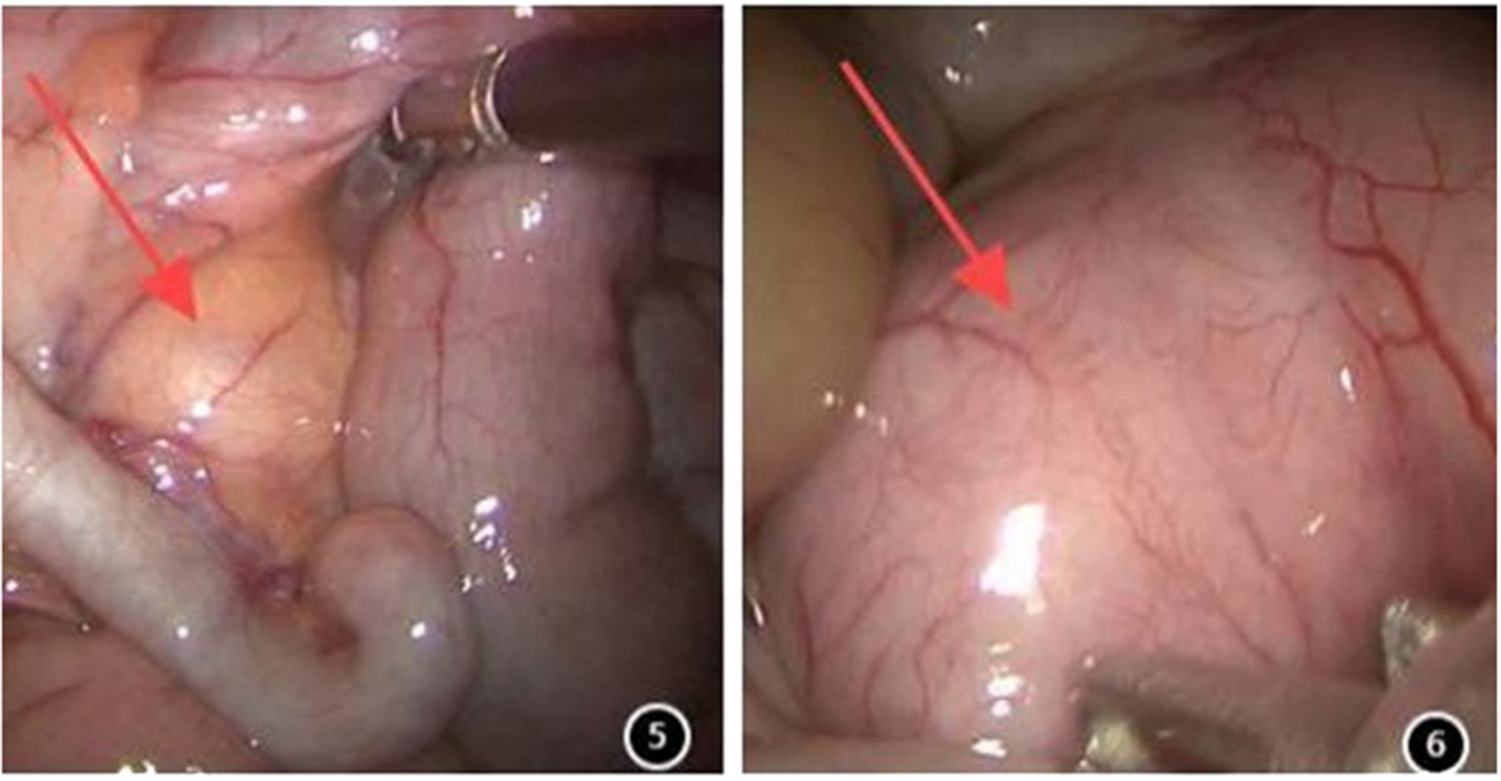
**(5,6)** Images of intestinal duplication malformations under laparoscopy.

Ninety children [female: 43, 47.8%; male: 47, 52.2%; median age: 21.0 months (IQR: 6.4–48.0 months)] underwent surgery. Out of 90 cases, 59 (65.56%) presented abdominal pain symptoms, 19 (21.11%) displayed intestinal obstruction symptoms, and 12 (13.33%) were asymptomatic and identified incidentally. All patients underwent outpatient ultrasound examination, which indicated an intestine duplication abnormality (Figures 1, 2), followed by enhanced abdominal CT (Figures 3, 4) conducted upon admission to confirm the diagnosis. Laparoscopic exploration was performed after surgical contraindications were excluded.

### 2.2 Laparoscopic surgical method

All the children underwent transumbilical single-site laparoscopic-assisted resection of intestinal duplication cysts. Gastric tubes and urinary catheters were placed preoperatively. The procedure began with a longitudinal incision approximately 1.5 cm in length at the umbilicus. The skin and subcutaneous tissues were incised sequentially. A single multi-channel port or individual 5 mm trocars were placed through the umbilical incision to establish CO_2_ pneumoperitoneum. A laparoscopic grasper was inserted through an operative port. Laparoscopic exploration was then performed to identify the lesion (Figures 5 and 6). The abdominal cavity was systematically inspected. Identification of a mass, adhesions, or significant congestion and edema of the bowel prompted focused examination of that area, as it frequently corresponded to the lesion site. If the lesion was not readily apparent, retrograde exploration of the small intestine commenced from the ileocecal region. Once located, the duplication cyst was grasped with an absorbable suture for traction. Subsequently, the umbilical port(s) and laparoscope were removed. The fascial (muscle layer and peritoneum) incision was extended under direct vision. The mass along with the affected bowel segment was then exteriorized through the umbilical incision. Depending on the cyst's size and location, either cyst excision (enterotomy and mucosal stripping) or bowel resection with primary anastomosis was performed. The resected specimen was sent for pathological examination.

## 3 Results

All 90 children underwent the surgery endoscopically, and none required conversion to laparotomy. The operative duration was 45–95 min. Among them, 74 patients underwent resection of the duplication malformation, whereas 16 patients underwent bowel resection with anastomosis. Of the latter, 5 underwent resection and anastomosis involving the terminal ileum, with the anastomosis situated 1.5–2 cm proximal to the ileocecal junction. No postoperative complications, including surgical site infection, anastomotic leak, anastomotic stenosis, adhesive bowel obstruction, or incisional hernia, occurred. Pathological examination of the surgical specimens confirmed intestinal duplication malformation in all cases.

All patients received perioperative antibiotics, hemostatic agents, and intravenous fluid resuscitation. Oral intake was resumed 1–4 days postoperatively, transitioned to semi-liquid then regular diet within 3–5 days, and discharge occurred 5–14 days after surgery. Prior to discharge, hemoglobin, biochemical parameters, and abdominal ultrasound were reassessed based on the children's clinical status. Following confirmation of normal results, the children were discharged. The mean length of hospitalization was 8.8 days. All patients attended a follow-up outpatient visit one month postoperatively, and no surgical site infections were noted. They reported no discomfort while tolerating a normal diet without spicy or irritating foods. Patients were followed for 3 months to 3 years. No late complications occurred, including surgical site infection, suture reaction, hypertrophic scar formation, or adhesive bowel obstruction. The surgical incisions healed well, were inconspicuous, and had an aesthetically pleasing appearance.

## 4 Discussion

Intestinal duplication malformations are uncommon congenital anomalies of the digestive tract in children, primarily located in the small intestine, particularly the terminal ileum, with an incidence rate of roughly 1/4,500 (3, 4). Clinically, there are generally no specific symptoms, with main manifestations including abdominal pain, intestinal obstruction, abdominal masses, cysts, and gastrointestinal bleeding (5, 6). These malformations may also precipitate intussusception (7), exacerbating intestinal injury and endangering the health and lives of children. Therefore, improving the diagnosis and treatment of intestinal duplication malformations in children is highly significant.

Intestinal duplication malformations may contain ectopic tissues such as ectopic gastric mucosa, pancreatic tissue, or colonic mucosa, which is one of the reasons for complications associated with intestinal duplication cysts. In addition, intussusception resulting from the lead point effect of the duplication itself is also a common presentation requiring medical attention. The non-specific symptoms are often confused with acute appendicitis, Meckel's diverticulum, mesenteric cysts, ovarian cysts, acute gastroenteritis, inflammatory bowel disease, tumors, or other acute abdominal conditions (8, 9), resulting in a relatively high rate of preoperative misdiagnosis and missed diagnosis. Currently, diagnostic modalities such as ultrasound, abdominal CT, 99mTc scanning, gastroscopy, capsule endoscopy, MR, and various other examinations are employed for the preoperative diagnosis of intestinal duplication malformations. However, all these diagnostic approaches have false-positives and false-negative rates. Therefore, for children suspected with intestinal duplication malformations, surgical exploration remains advisable for clear diagnosis and corresponding treatment. In clinical practice, abdominal ultrasound and CT are the most commonly utilized diagnostic tools (10, 11). Among them, ultrasound demonstrates a high detection rate for intestinal duplication malformations in children. Compared with other imaging modalities, it offers advantages such as strong operability, simplicity, rapidity, absence of radiation, non-invasiveness, and the capacity to be repeated. This diagnostic method is highly effective and reliable, serving as the primary approach for preoperative diagnosis and postoperative follow-up of intestinal duplication abnormalities in children (12). Despite high suspicion based on imaging findings, confirmatory diagnosis typically requires surgical exploration and subsequent histopathological examination.

Surgical intervention is the primary treatment for intestinal duplication malformations. Surgical methods include traditional open surgery and laparoscopic approaches. Although traditional open surgery is technically straightforward, it requires a large incision, causes substantial trauma, and results in significant postoperative pain in pediatric patients. The incidence of complications such as surgical site infection and fat necrosis is relatively high. Moreover, the extensive manipulation of the digestive tract delays postoperative recovery of intestinal peristalsis, increasing the risk of intestinal adhesions and obstruction. Additionally, it leaves prominent abdominal scars, compromising cosmesis. Therefore, it is rarely performed currently, reserved only for cases with poor general condition, significant abdominal distension contraindicating pneumoperitoneum, or concurrent intra-abdominal pathologies requiring intervention.

In contrast, laparoscopic surgery has been widely accepted and applied owing to its benefits of less trauma, less postoperative pain, faster recovery, and aesthetic incisions (13). Currently, we use single-site laparoscopic-assisted resection of intestinal duplication malformations through the umbilicus. Under this technique, a two-port laparoscopic exploration is first conducted through the umbilicus. After confirming the diagnosis, the affected bowel segment is resected and anastomosed through an extended umbilical incision. This method leverages the umbilicus's natural concavity, resulting in well-concealed incisions with excellent cosmesis, minimized abdominal trauma, accelerated recovery, and reduced complications—consistent with reported benefits of single-port laparoscopy (14).

In older children, extraction of the relatively fixed ileocecal region may be challenging. If direct extraction is difficult, the paracolic peritoneum can be dissected first to facilitate exteriorization through the umbilical incision. Following exteriorization of the malformation, complete excision is the optimal approach. Successful cystectomy requires proficient coordination between the surgeon and assistant. The assistant must provide appropriate traction and counter-traction to enable the surgeon to perform the resection effectively, while exercising meticulous care to avoid mucosal injury.

Smaller malformations should be excised completely with preservation of integrity. In cases with significant deformities, the cyst may be incised, allowing for meticulous identification of the margins from within prior to excision. Children with challenging abnormalities necessitate intestinal resection and anastomosis. Previous experience indicates that abnormalities in the ileocecal region should be addressed through excision of the ileocecal area and subsequent ileocolic anastomosis. However, the ileocecal region, comprising the terminal ileum, ileocecal valve, and cecum, performs critical physiological functions: the ileocecal valve serves as a unidirectional barrier that inhibits the retrograde movement of small intestinal contents and bacterial contamination while decelerating the filling of the colon and rectum; the cecum is involved in the reabsorption of vitamin B12, electrolytes, fatty acids, bile salts, and water (15). Consequently, preserving the ileocecal region is crucial to reduce postoperative complications like bacterial translocation, diarrhea, and electrolyte disturbances. This series reports five children who underwent terminal ileal resection and anastomosis without ileocecal resection, with no anastomotic leaks observed. This supports recent evidence advocating maximal preservation of ileocecal function (13). Therefore, for children with difficult-to-remove malformations, one-stage resection and anastomosis of the affected intestinal segment (rather than ileocecal resection) is safe and reliable, avoiding complications associated with ileocecal resection and the morbidity of enterostomy. The study's sample size is limited, necessitating further clinical data for validation.

In conclusion, this study, combined with literature reports (13–15), confirms that transumbilical single-port laparoscopic-assisted resection of intestinal duplication malformation is safe and effective, with minimal complications and excellent cosmetic results. It represents a preferred surgical approach for pediatric intestinal duplication malformation.
